# Comparison of clinical outcomes with proximal femoral nail anti-rotation versus InterTAN nail for intertrochanteric femoral fractures: a meta-analysis

**DOI:** 10.1186/s13018-020-02031-8

**Published:** 2020-10-29

**Authors:** Wei Liu, Jie Liu, Guangrong Ji

**Affiliations:** 1 Department of Orthopaedics, Xiang’an Hospital, School of Medicine, Xiamen University, Xiamen, 361102 China; 2 Graduate School, Tianjin Medical University, Tianjin, 300070 China

**Keywords:** Unstable femoral intertrochanteric fractures, Proximal femoral nail anti-rotation, InterTAN nail, Meta-analysis

## Abstract

**Background:**

A meta-analysis to access the pros and cons of proximal femoral nail anti-rotation (PFNA) versus InterTAN nail for intertrochanteric femoral fractures including available evidence extracted from literature.

**Methods:**

According to the Cochrane systemic analysis method, randomized control trials (RCTs) and retrospective comparative observational studies which were related to the comparison of PFNA and InterTAN nail in the treatment of the elderly with intertrochanteric fractures were retrieved. Data were independently extracted from the included studies by two reviewers and analyzed using *RevMan 5.3*, and the quality of the studies was assessed.

**Results:**

Two RCTs and seven observational studies were recruited, which consisted of 681 patients with PFNA and 651 patients with InterTAN nail. The meta-analyses showed no significant differences between the two approaches on Harris Hip Score, operation time, blood loss, time to union, mean hospital stay, union problems, intraoperative complications, hematoma, infection, and other complications in both RCTs and observational studies. In terms of other outcomes, for the RCTs, results showed that there were shorter tip–apex distance and reduced pain at thigh or hip in InterTAN nail than in PFNA; however, InterTAN nail was not superior to PFNA in cutout, reoperation, and femoral shaft fracture; for observational studies, the risk of the screw migration (RR = 5.13, 95%CI [1.33,19.75], *P* = 0.02), cutout (RR = 3.26, 95%CI [1.64,6.47], *P* = 0.0008), the varus collapse of the femoral head (RR = 7.19, 95%CI [2.18,23.76], *P* = 0.001), femoral shaft fracture (RR = 5.73, 95%CI [2.24,14.65], *P* = 0.0003) treated by InterTAN nail were significantly decreased, compared with those by PFNA; however, no significant differences were observed in the aspects of tip–apex distance and pain at thigh or hip between these two groups.

**Conclusion:**

Analysis of a large number of relevant clinical indicators available shows that InterTAN nail has better clinical manifestation than PFNA in treating unstable femoral intertrochanteric fractures.

**Supplementary Information:**

The online version contains supplementary material available at 10.1186/s13018-020-02031-8.

## Background

In the aged population, intertrochanteric fractures, leading to severe functional impairments, are one of the most frequent fractures; besides, it has become a severe health issue due to the rapid increase in the aged population in recent years [1–4]. Some studies reported that the annual incidence of hip fractures exceeds 320,000 cases in North America, and by 2050, this number is projected to rise to 6 million with an average annual mortality rate of more than 20% for hip fractures and intertrochanteric fractures [5].

Surgery is the mainstay of the treatment of unstable femoral intertrochanteric fractures, mainly including extramedullary fixation and intramedullary fixation. Dynamic hip screws in extramedullary fixation have been widely used and were considered as the gold standard for extracapsular fractures previously [4]. However, multiple meta-analyses have shown that intramedullary fixation, compared with extramedullary fixation, could benefit the patient in terms of reduced risk of implant failure and reoperation, as well as improved functional scores [6–8]. PFNA is a kind of intramedullary fixation with a large area of spiral blade, which can achieve tighter bone compaction and femur alignment than traditional screws, providing optimal anchoring and stability for intertrochanteric fractures [2, 9–11]. In addition, InterTAN nail, as another type of intramedullary fixation, uses an integrated interlocking lag nail system that can better limit the movement of the femoral head and can effectively avoid the collapse of the femoral head [6, 12–18].

However, the data on the comparison of the clinical outcomes of PFNA and InterTAN nail are so far insufficient and even controversy. For example, Seyhan et al. stated that stronger hold of the femoral head by the hip screw and efficacious compression play major roles in the outcome, as less screw backup and femoral shortening were seen with the InterTAN group than with the PFNA group [14]. In contrast, a previous meta-analysis conducted by Ma et al. reported that the clinical outcomes including cutout and femoral fractures are in favor of the PFNA group when compared to the InterTAN nail [19]. Accordingly, the current meta-analysis was conducted to comprehensively compared clinical outcomes of two techniques and further determines the optimal treatment for patients with intertrochanteric fractures.

## Methods

### Search strategy

**Fig. 1:**
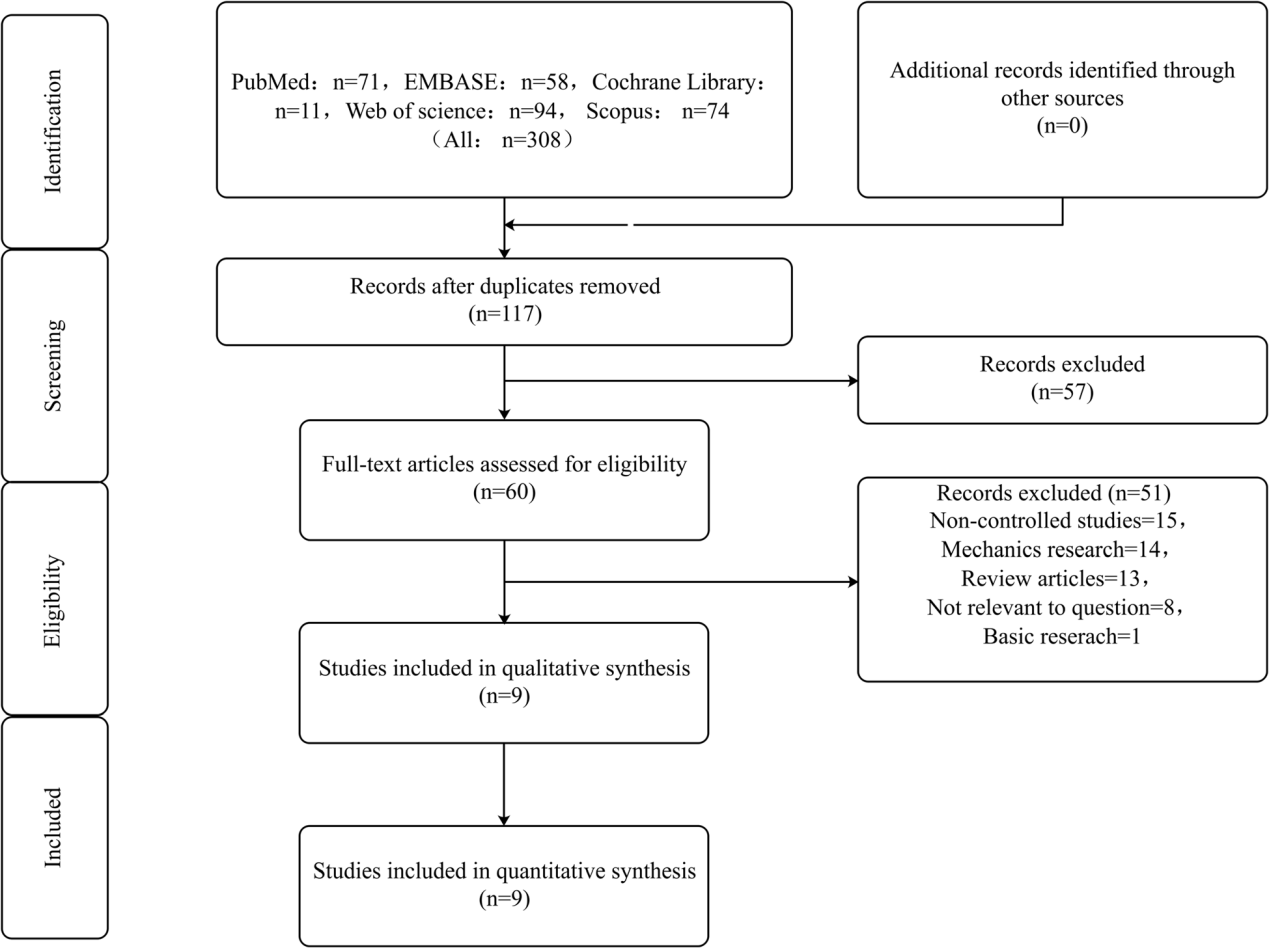
Flow diagram of literature selection

According to the search strategy of the Cochrane Collaboration, the following databases were searched for related articles published before February 2020: MEDLINE, Web of Science, Scopus, Cochrane Central Register of Controlled Trials (CENTRAL), EMBASE; the references listed in relevant literature were further screened to ensure the comprehensiveness and diversity of the review. The search terms are presented in Supplemental List 1, and details of the selection process are outlined in a flow chart (Fig. 1).

### Inclusion and exclusion criteria

All of the titles and abstracts were screened by two authors independently using the PICO framework as follows: (1) population: individuals with intertrochanteric fractures or pertrochanteric fractures; (2) intervention: PFNA; (3) comparator: InterTAN nails; (4) outcomes: Harris Hip Score (HHS), blood loss, cutout, tip–apex distance, operation time, reoperation, femoral shaft fractures, infection, intraoperative complications, length of hospital stay, hematoma, pain at thigh or hip, time to union, union problems, femoral head abnormalities, screw migration, and other complications; and (5) study design: several studies (prospective, randomized controlled trials (RCTs) or comparative observational studies published in any language) were eligible for inclusion. The present study included clinical trials with at least one main clinical outcome described above, even though the follow-up in some studies was less than 12 months. Nevertheless, review articles, biomechanical researches, trials in animals, uncontrolled experiments, and duplicate or multiple publications of the same study were excluded. The reviewers resolved any disagreements and differences through discussion and consensus or, if required, by consulting the corresponding author.

### Data extraction

Relevant data in the eligible studies were independently extracted by the two authors using predefined data extraction sheets, then cross-checked; besides, the reviewers resolved any differences by consensus. Data mainly included the main authors of the study, year of publication, sample size, patient baseline characteristics (e.g., gender and age), fracture types of patients, the follow-up time, and the reported outcomes, which were summarized in Table 1.

**Table 1:** The characteristics of included studies

Study, year	PFNA^a^/IT^b^						Length of follow-up (months)	Type of study^e,f^
	Sample size	Age (years)	Gender (% male)	Fracture type (number)				
				AO/OTA-A1^c^	AO/OTA-A2	AO/OTA-A3		
Duramaz, 2019 [20]	100/86	PFNAII = 61.0 ± 16.6 IT = 61.5 ± 15.8	43.6^d^	28/34	49/32	23/20	25.9	RS
Gavaskar, 2018 [21]	50/50	PFNAII = 78 ± 8 IT = 77 ± 7	42.0/42.0	0/0	31/31	19/19	12	RS
Seyhan, 2015 [14]	43/32	PFNA = 75.9 ± 13.77 IT = 75.3 ± 13.52	25.6/25.0	44142	16/13	44181	19.4 (mean)	RCT
Wang, 2013 [22]	36/20	PFNAII = 76.8 ± 9.5 IT = 73.5 ± 11.3	47.2/55	7/2	26/13	3/5	4.1 (mean)	RS
Yu, 2016 [23]	72/75	PFNAII = 74.2 ± 9.1 IT = 75.2 ± 8.8	44.4/46.7	0/0	35/40	37/35	20 (mean)	RS
Zehir, 2015 [24]	96/102	PFNA = 77.2 ± 6.8 IT = 76.8 ± 6.7	38.5/38.2	0/0	92/93	43930	16.06/16.00	RS
Zhang, 2013 [25]	56/57	PFNAII = 72.4 ± 8.7 IT = 72.9 ± 7.6	33.9/40.4	0/0	45/45	44147	18.36	RCT
Zhang, 2017a [26]	88/86	PFNAII = 74.6 ± 6.3 IT = 72.7 ± 7.6	38.6/34.9	42/37	46/49	0/0	41.51/40.84	RS
Zhang, 2017b [27]	139/144	PFNA/IT = 76.1^d^	38.1/44.4	0/0	139/144	0/0	39.1/38.7	RS

Data are presented as *n* or mean ± standard deviation

^a^Proximal femoral nail anti-rotation

^b^InterTAN nail

^c^Arbeitsge-meinschaft für Osteosynthesefragen/Orthopaedic Trauma Association

^d^Population parameters

^e^Retrospective study

^f^Randomized controlled trial

### Statistical analysis

A meta-analysis was conducted using *RevMan 5.3 software*. The relative risk (RR) was used for evaluating dichotomous outcomes such as cutout, tip–apex distance, reoperation, femoral shaft fractures, infection, intraoperative complications, hematoma, pain at thigh or hip, union problems, femoral head abnormalities, screw migration. For continuous data, and the mean difference (MD) with a 95% confidence interval was recorded, and *P* value below 0.05 was deemed as statistically significant. The heterogeneity test was performed on the studies using *P* and *I*^2^ statistics; besides, a fixed-effects model was applied where the heterogeneity between studies was not substantial (*I*^2^ < 50% and *P* > 0.1); otherwise, a random-effects model was selected(50% ≤ *I*^2^ ≤ 100%). Meanwhile, subgroup analysis was applied according to different types of included studies. A combined analysis was used to make full use of the available data when heterogeneity between studies is not significant. Moreover, a sensitivity analysis was performed by iteratively excluding one study at a time to confirm the robustness of the results. However, funnel plot was not reported due to insufficient literature included.

## Results

### Baseline characteristics of included studies

The search yielded 308 potentially relevant reports, and 117 remained after the duplicate articles were deleted, of which 57 were excluded by preliminary screening; further careful screening of the full text excluded 51 articles leaving 9 for detailed evaluation. Nine studies (*n* = 1332 patients) are published between January 2013 and December 2019 that fulfilled the inclusion and exclusion criteria, including 2 RCTs and 7 observational studies [14, 20–27]. A total of 1332 patients were relatively evenly distributed in PFNA (*n* = 681 patients) and InterTAN nail groups (*n* = 651 patients), and the proportion of the two surgical procedures in A1, A2, and A3 fractures is 88/80, 479/460, and 113/112, respectively. All of the studies were followed up for at least 12 months, except for ref [28]. Of these, five studies only included patients with unstable intertrochanteric fractures (e.g., AO/OTA classification A2–A3 fractures), and the other 4 had a mixed type of intertrochanteric fractures. All RCTs were classified as unclear risk of bias since no blind methods were reported in included studies, and most observational studies were considered adequate quality based on the GRACE checklist [29].

### Clinical outcomes

#### Primary outcome

##### HHS

**Fig. 2:**
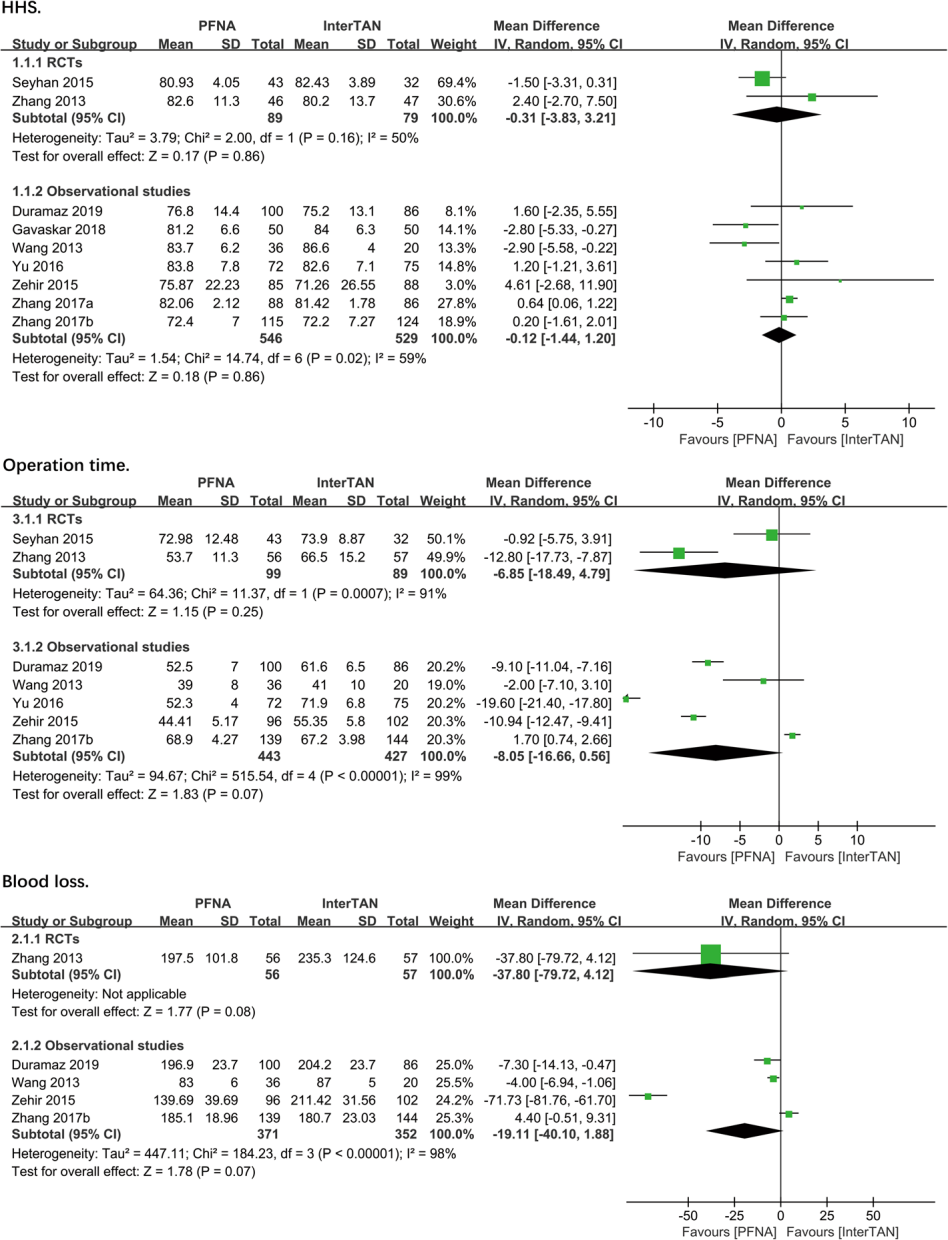
A forest plot diagram showed HHS, operation time, and blood loss

HHS was recorded in all 9 studies (*n* = 1243). Overall, there were no significant differences between PFNA and InterTAN nails in both RCTs (MD = − 0.31, 95%CI [− 3.83, 3.21], *P* = 0.86; Fig. 2) and observational studies (MD = − 0.12, 95%CI [− 1.44, 1.20], *P* = 0.86; Fig. 2) using the random-effects model as a result of moderate heterogeneity in two subgroups (RCTS: chi^2^ = 2.00, df = 1, *P* = 0.16, *I*^2^ = 50%; observational studies: chi^2^ = 14.774, df = 6, *P* = 0.02, *I*^2^ = 59%). To verify the robustness and reliability of the present results, a sensitivity analysis was performed by presenting similarly heterogeneity before and after each of the study removed.

#### Secondary outcomes

##### Operation time

Operation time was reported in 7 studies (*n* = 1058 patients). No significant differences were observed in PFNA versus InterTAN nails in both RCTs (MD = − 6.85, 95%CI [− 18.49, 4.79], *P* = 0.25; Fig. 2) and observational studies (MD = − 8.05, 95%CI [− 16.66, 0.56], *P* = 0.07; Fig. 2) using the random-effects model with the statistical heterogeneity (RCTS: chi^2^ = 11.37, df = 1, *P* = 0.0007, *I*^2^ = 91%; observational studies: chi^2^ = 515.4, df = 4, *P* < 0.00001, *I*^2^ = 99%) that may be related to different measurement methods.

##### Blood loss

Five studies consisting of 1 RCT and 4 observational studies showed the outcome of blood loss. No significant differences were observed in PFNA versus InterTAN in both RCT (MD = − 37.80, 95%CI [− 79.72, 4.12], *P* = 0.08; Fig. 2) and observational studies (MD = − 19.11, 95%CI [− 40.10, 1.88], *P* = 0.07; Fig. 2) using the random-effects model due to the statistical heterogeneity (observational studies: chi^2^ = 184.23, df = 3, *P* < 0.00001, *I*^2^ = 98%).

##### Tip–apex distance

**Fig. 3:**
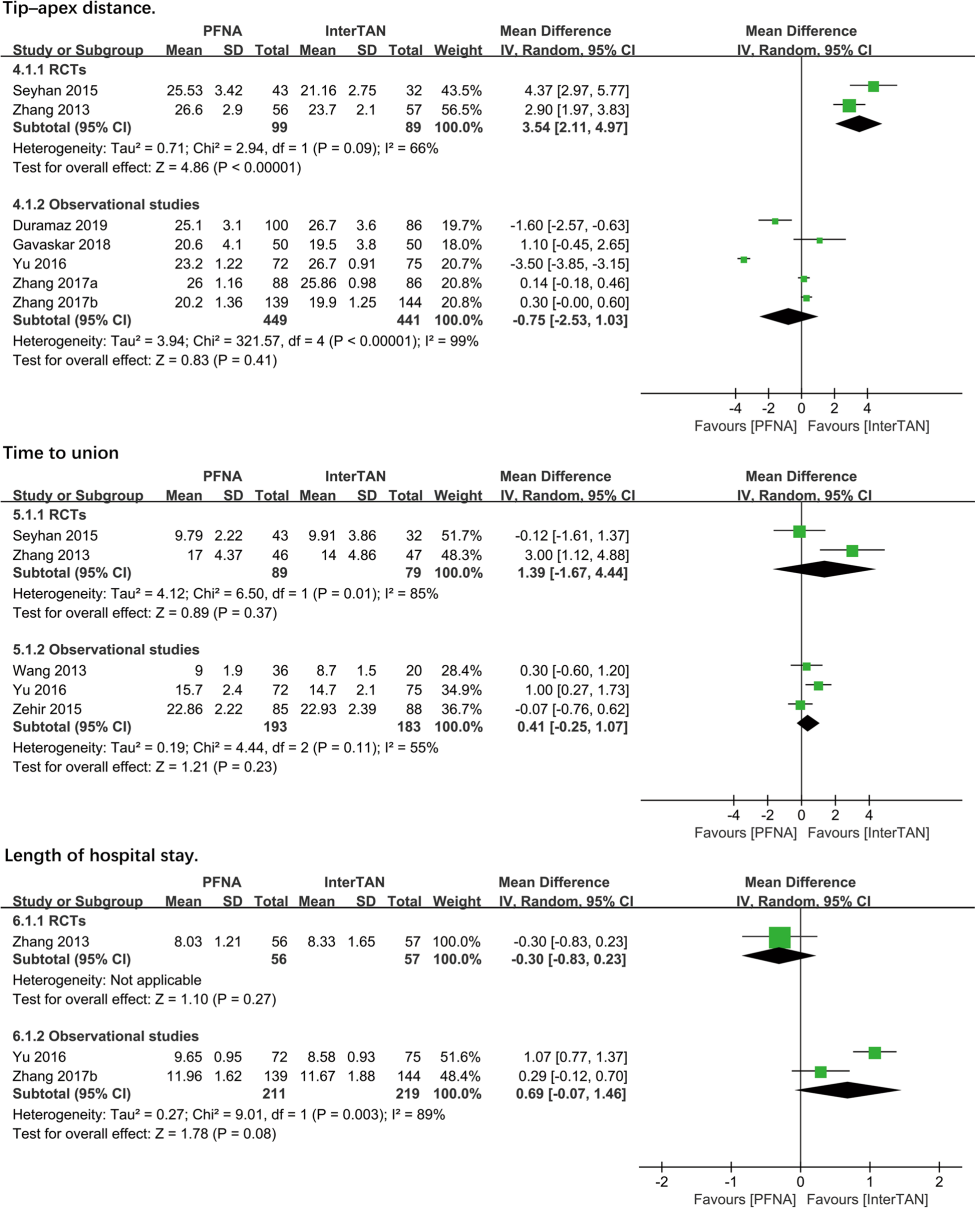
A forest plot diagram showed TAD, time to union, and length of hospital stay

Data from 2 RCTs and 5 observational studies (*n* = 1078 patients) showed tip–apex distance postoperatively. Subgroup analysis showed significant difference in the RCTs subgroup (MD = 3.54, 95%CI [2.11, 4.97], *P* < 0.00001; Fig. 3) but not in the observational studies subgroup. (MD = − 0.75, 95%CI [− 2.53, 1.03], *P* = 0.41; Fig. 3) using the random-effects model with the statistical heterogeneity (RCTS: chi^2^ = 2.94, df = 1, *P* = 0.09, *I*^2^ = 66%; observational studies: chi^2^ = 321.57, df = 4, *P* < 0.00001, *I*^2^ = 99%). A combined analysis of subgroups was not performed due to the relatively large heterogeneity of subgroups.

##### Time to union

Data from 5 studies (*n* = 544 patients) reported time to union. No significant differences were found between PFNA and InterTAN nails through subgroup analysis in both RCTs (MD = 1.39, 95%CI [− 1.67, 4.44], *P* = 0.37; Fig. 3) and observational studies (MD = 0.41, 95%CI [− 0.25, 1.07], *P* = 0.23; Fig. 3) using the random-effects model as a result of moderate to high heterogeneity in two subgroups (RCTS: chi^2^ = 6.50, df = 1, *P* = 0.01, *I*^2^ = 85%; observational studies: chi^2^ = 4.44, df = 2, *P* = 0.11, *I*^2^ = 55%).

##### Length of hospital stay

One RCT and 2 observational studies (*n* = 543 patients) showed the mean hospital stay. No significant differences were found between PFNA and InterTAN nails through subgroup analysis in both RCTs (RR = − 0.30, 95%CI [− 0.83, 0.23], *P* = 0.27; Fig. 3) and observational studies (RR = 0.69, 95%CI [− 0.07, 1.46], *P* = 0.08; Fig. 3). The random-effects model was adopted, as the heterogeneity analysis had shown a significant difference (observational studies: chi^2^ = 9.01, df = 1, *P* = 0.003, *I*^2^ = 89%), which may be related to the different health status of the patients in each group.

##### Cutout

**Fig. 4:**
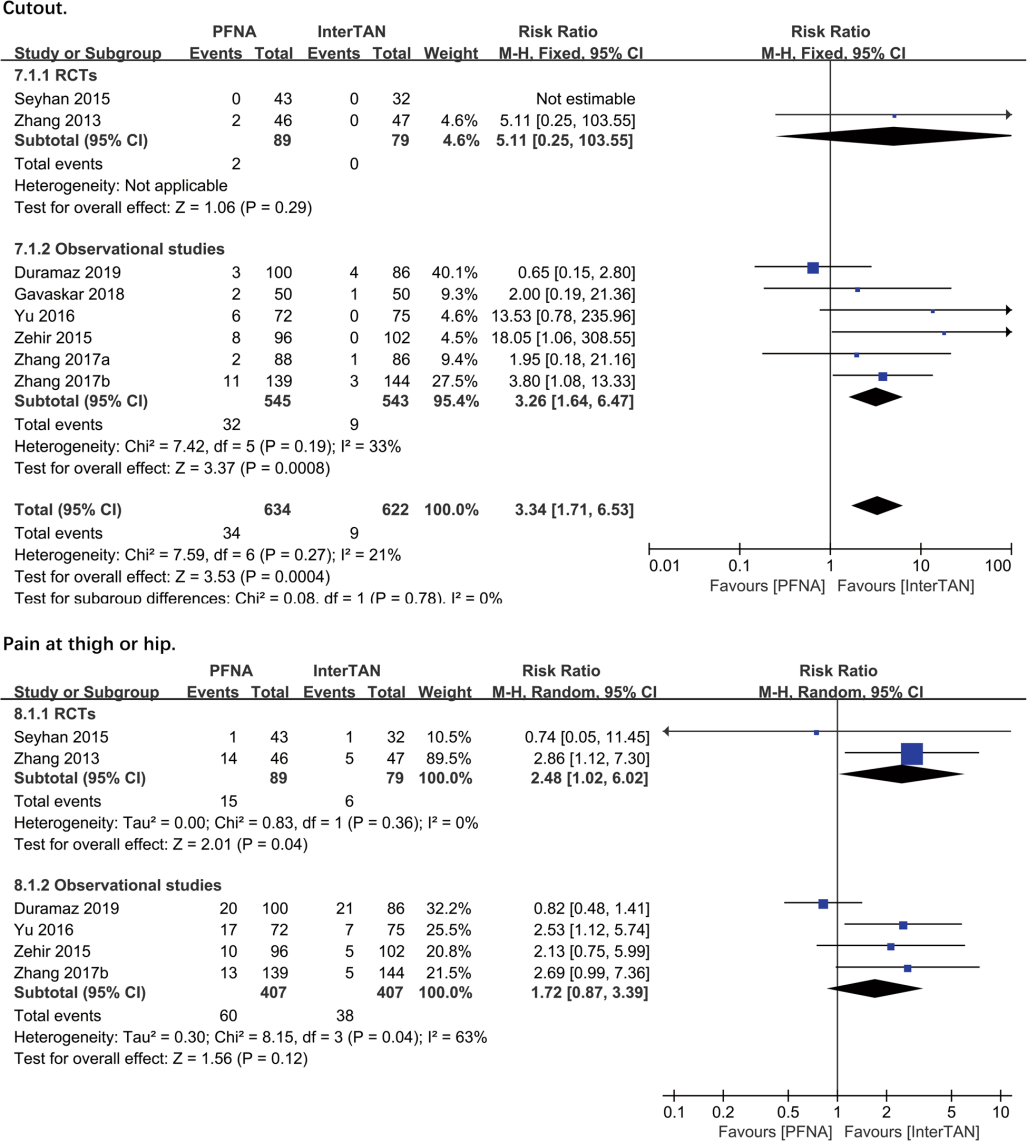
A forest plot diagram showed cutout and pain at thigh or hip

A total of 8 studies with 1256 patients reported the outcome of the cutout. It showed no significant differences comparing PFNA with InterTAN for the RCTs subgroup, However, significant difference was present in the observational subgroup (RR = 3.26, 95%CI [1.64, 6.47], *P* = 0.0008; Fig. 4). A combined analysis was performed and the differential effect between PFNA and InterTAN remains significant (RR = 3.34, 95%CI [1.71, 6.53], *P* = 0.0004; Fig. 4). The fixed-effects model was used as the heterogeneity analysis had not shown a significant difference (observational studies: chi^2^ = 7.42, df = 5, *P* = 0.19, *I*^2^ = 33%; total: chi^2^ = 7.59, df = 6, *P* = 0.27, *I*^2^ = 21%).

##### Pain at thigh or hip

Six studies (*n* = 1150 patients) reported the outcome of pain at the thigh or hip. In subgroup analysis, a significant difference existed in the RCTs subgroup (RR = 2.48, 95%CI [1.02, 6.02], *P* = 0.04; Fig. 4), but not in the other subgroup (RR = 1.72, 95%CI [0.87, 3.39], *P* = 0.12; Fig. 4). According to the magnitude of the heterogeneity value, the former used the fixed-effects model, while the latter adopted the random-effects model (RCTS: chi^2^ = 0.83, df = 1, *P* = 0.36, *I*^2^ = 0%; observational studies: chi^2^ = 8.15, df = 3, *P* = 0.04, *I*^2^ = 63%). Notably, Duramaz et al.’s [28] study was found to be the origin of the heterogeneity occurred in the observational studies subgroup when sensitivity analysis was performed, and *I*^2^ statistics changed from 63% to zero when the study was removed. Furthermore, significant differences were found in both subgroups, and combined analysis showed a significant difference (RR = 2.46, 95%CI [1.55, 3.91], *P* = 0.0001; Fig. 4) after the study was excluded.

##### Reoperation

**Fig. 5:**
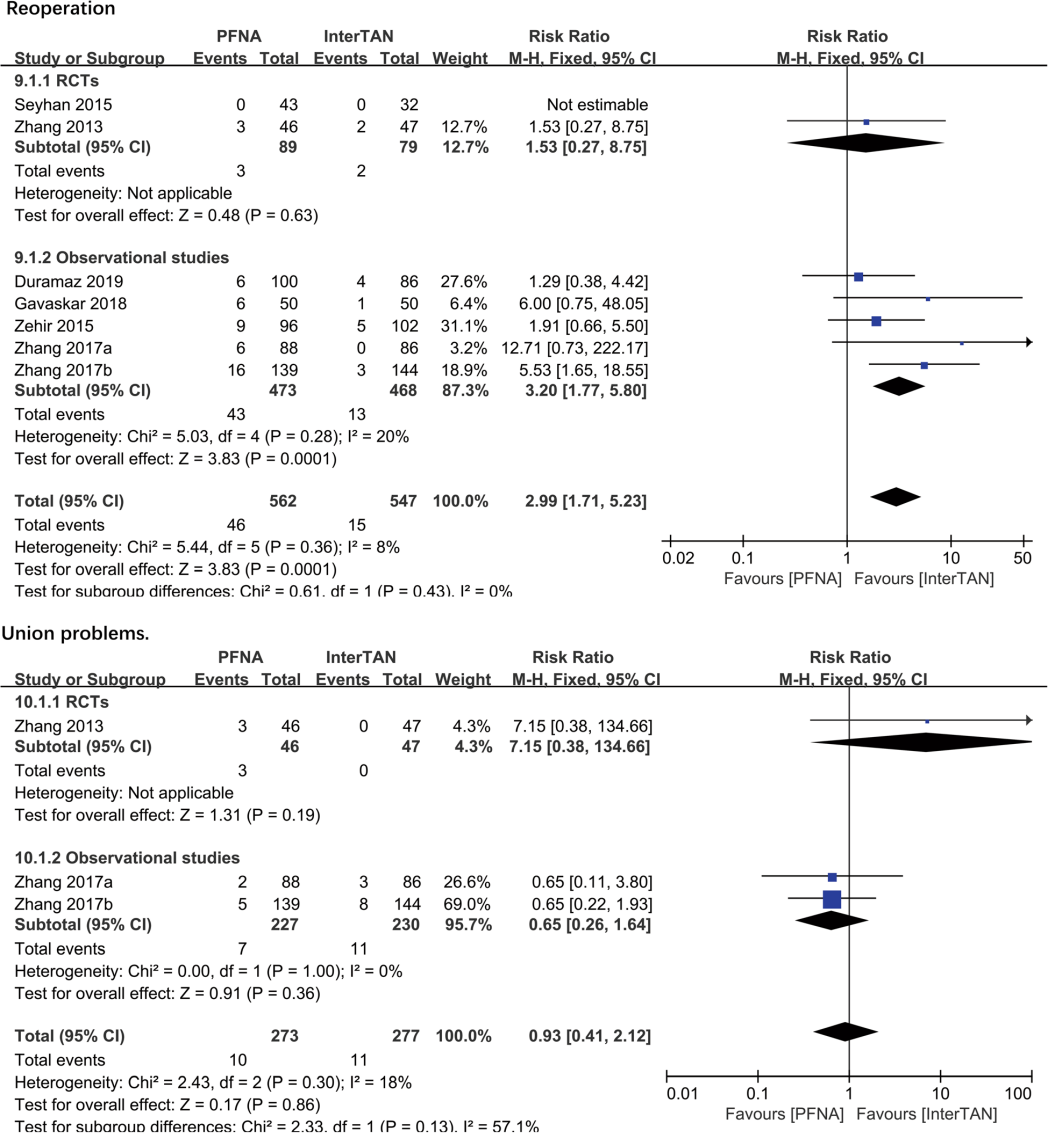
A forest plot diagram showed reoperation and union problems

Seven studies with 1109 patients reported on the reoperation. A significant difference existed in the observational subgroup (RR = 3.20, 95%CI [1.77, 5.80], *P* = 0.0001; Fig. 5) but not in the RCT subgroup (RR = 1.53, 95%CI [0.27, 8.75], *P* = 0.63; Fig. 5). The fixed-effects model and combined analysis were conducted due to the low heterogeneity and a significant difference (RR = 2.99, 95%CI [1.71, 5.23], *P* = 0.0001; Fig. 5) in total between PFNA and InterTAN was reported (observational studies: chi^2^ = 5.03, df = 4, *P* = 0.28, *I*^2^ = 20%; total: chi^2^ = 5.44, df = 5, *P* = 0.36, *I*^2^ = 8%).

##### Union problems

Three studies (*n* = 550 patients) reported on union problems consisted of delayed union, malunion, and nonunion. No significant difference was found between PFNA and InterTAN no matter in RCTs subgroup (RR = 7.15, 95%CI [0.38, 134.66], *P* = 0.19; Fig. 5) or observational subgroup (RR = 0.65, 95%CI [0.26, 1.64], *P* = 0.36; Fig. 5) or in combined analysis (RR = 0.93, 95%CI [0.41, 1.64], *P* = 0.86; Fig. 5). No significant heterogeneity was found, and then the results were synthesized using the fixed-effects model (observational studies: chi^2^ = 0.00, df = 1, *P* = 1.00, *I*^2^ = 0%; total: chi^2^ = 2.43, df = 2, *P* = 0.30, *I*^2^ = 18%).

##### Screw migration

**Fig. 6:**
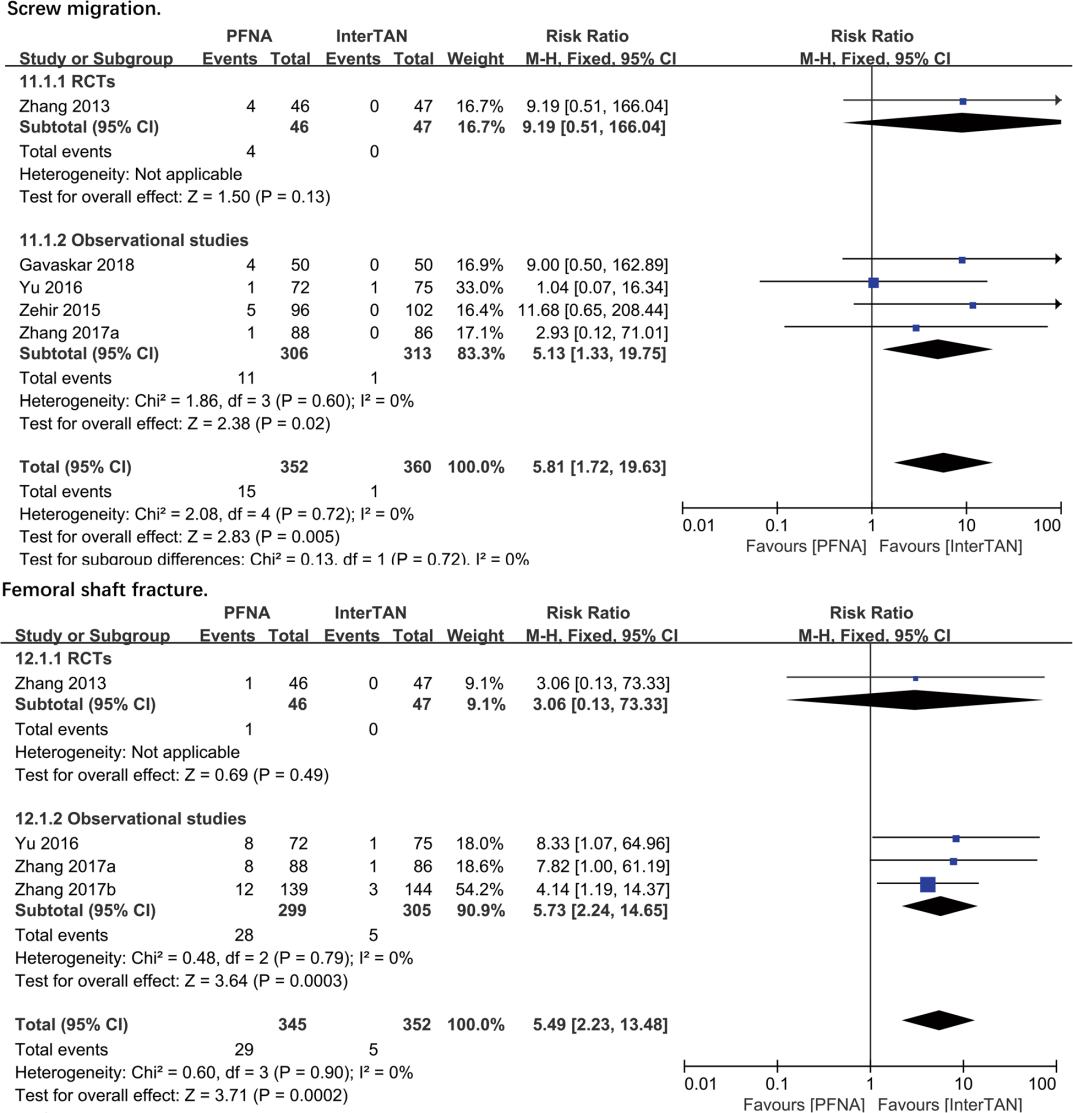
A forest plot diagram showed screw migration and femoral shaft fracture

Five studies (*n* = 712 patients) reported on the screw migration problems. A significant difference was found in both observational studies subgroup (RR = 5.13, 95%CI [1.33, 19.75], *P* = 0.02; Fig. 6) and combined analysis (RR = 5.81, 95%CI [1.72, 19.63], *P* = 0.005; Fig. 6). The fixed-effects model was performed to calculate a pooled effect as no significant heterogeneity was found (observational studies: chi^2^ = 1.86, df = 3, *P* = 0.60, *I*^2^ = 0%; total: chi^2^ = 2.08, df = 4, *P* = 0.72, *I*^2^ = 0%)

##### Femoral shaft fracture

Four studies (*n* = 697 patients) reported on the femoral shaft fracture outcome. Despite a significant difference (RR = 3.06, 95%CI [0.13, 73.33], *P* = 0.005; Fig. 6) was not found between PFNA and InterTAN in a single RCT study, a significant difference can be found in the other subgroup (RR = 5.73, 95%CI [2.24, 14.65], *P* = 0.0003; Fig. 6). Due to a lack of heterogeneity, a combined analysis was performed and a significant difference (RR = 5.49, 95%CI [2.23, 13.48], *P* = 0.0002; Fig. 6) between PFNA and InterTAN nail in total was observed. No significant heterogeneity was found, and then the current results were synthesized using the fixed-effects model (observational studies: chi^2^0.48, df = 2, *P* = 0.79, *I*^2^ = 0%; total: chi^2^ = 0.60, df = 3, *P* = 0.90, *I*^2^ = 0%).

##### Femoral head abnormalities

**Fig. 7:**
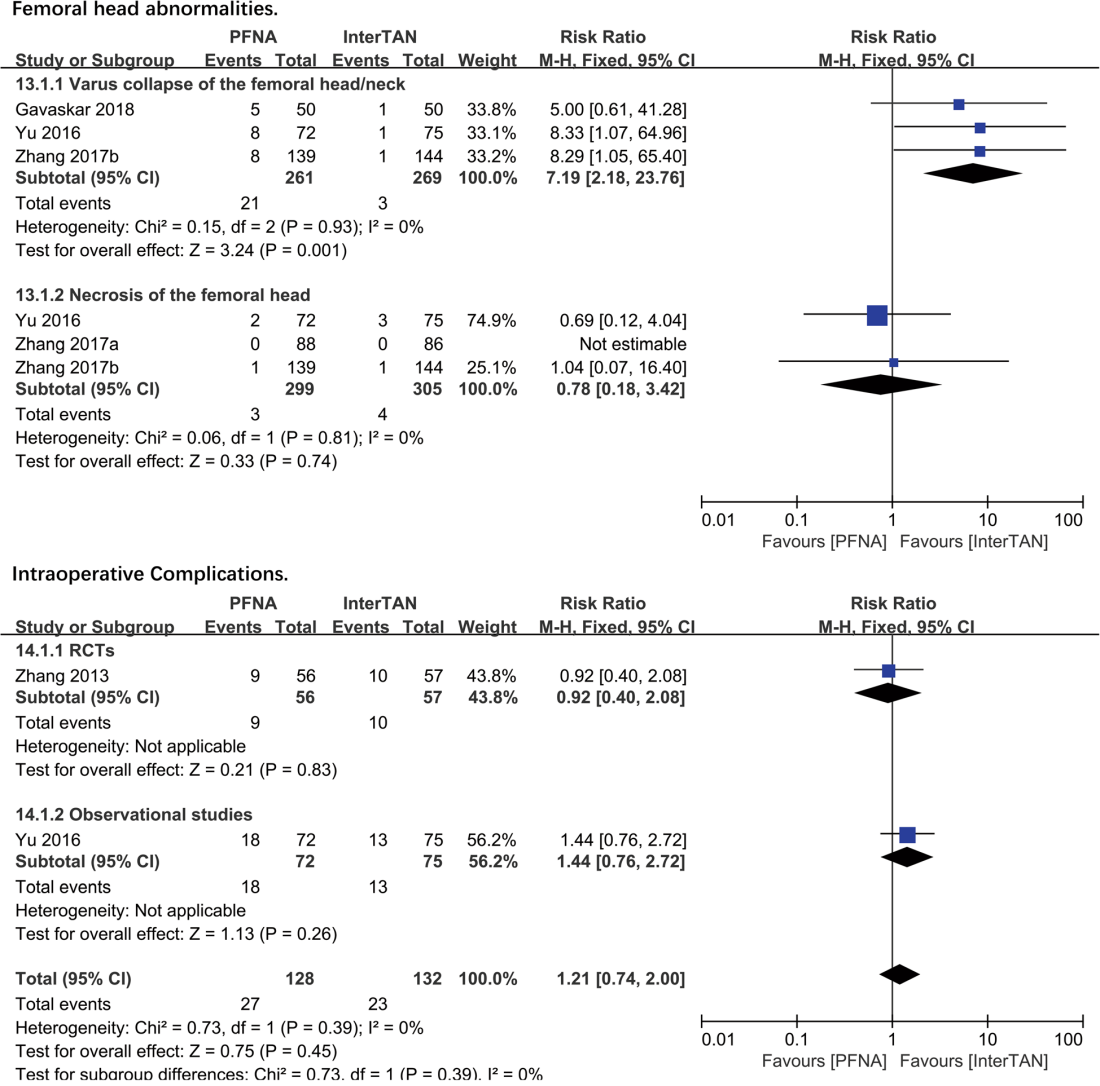
A forest plot diagram showed femoral shaft fracture and intraoperative complications

Femoral head abnormalities included the femoral head necrosis and the varus collapse of the femoral head/neck. Three studies with 530 patients reported the varus collapse of the femoral head/neck while 3 studies from the same or different teams described above with 604 patients reported on the necrosis of the femoral head. A subgroup analysis was used and a significant difference existed (RR = 7.19, 95%CI [2.18, 23.76], *P* = 0.001; Fig. 7) in PFNA and InterTAN referring to the rate of varus collapse of the femoral head/neck postoperatively, while no significant difference (RR = 0.78, 95%CI [0.18, 3.42], *P* = 0.74; Fig. 7) presented about the necrosis of the femoral head between the two kinds of the nail. The pooled effect was calculated by the fixed-effects model as no significant heterogeneity was found (RCTS: chi^2^ = 0.15, df = 2, *P* = 0.03, *I*^2^ = 0%; observational studies: chi^2^ = 0.06, df = 1, *P* = 0.81, *I*^2^ = 0%).

##### Intraoperative complications

Intraoperative complications included fractures that occurred in the greater trochanter, lateral cortex, or femoral shaft, changes in distal interlocking position, and penetration of the trochanter or femoral head. Overall, 1 RCT and 1 observational study reported on the intraoperative complications, including 260 patients. No significant difference was found in PFNA and InterTAN nail in either RCTs (RR = 0.92, 95%CI [0.40, 2.08], *P* = 0.83; Fig. 7) or observational studies (RR = 1.44, 95%CI [0.76,2.72], *P* = 0.26; Fig. 7). A combined analysis showed no significant difference (RR = 1.21, 95%CI [0.74, 2.00], *P* = 0.45; Fig. 7) between PFNA and InterTAN nail in intraoperative complications. The fixed-effects model was performed, as no heterogeneity was shown in heterogeneity analysis (total: chi^2^ = 0.73, df = 1, *P* = 0.39, *I*^2^ = 0%).

##### Hematoma

**Fig. 8:**
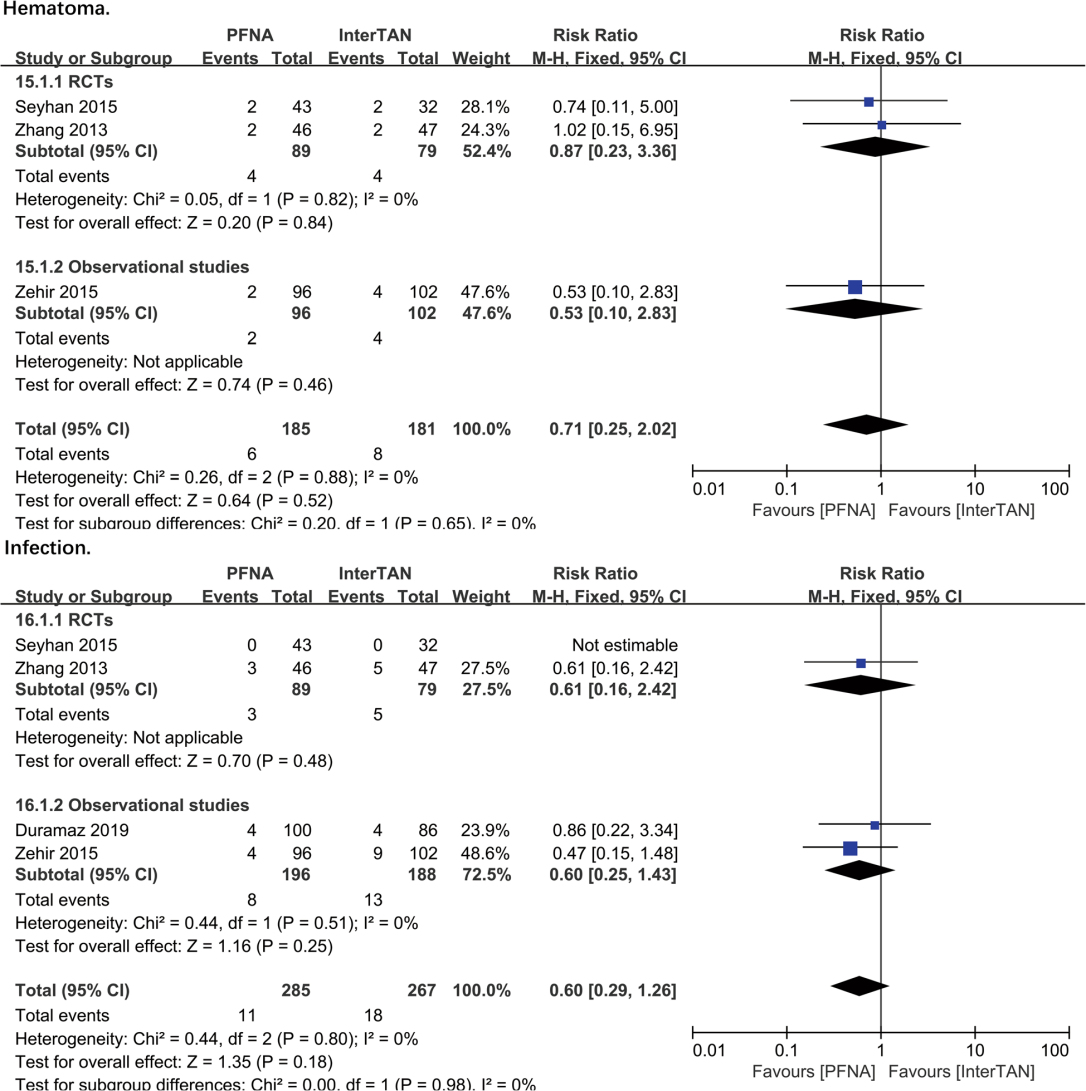
A forest plot diagram showed hematoma and infection

The outcome of hematoma was reported in 3 studies (*n* = 366 patients). No significant difference was found in PFNA and InterTAN in subgroups of RCTs (RR = 0.87, 95%CI [0.23, 3.36], *P* = 0.84; Fig. 8) and observational studies (RR = 0.53, 95%CI [0.10, 2.83], *P* = 0.46; Fig. 8). Correspondingly, the combined analysis showed no significant difference (RR = 0.71, 95%CI [0.25, 2.02], *P* = 0.52; Fig. 8). The fixed-effects model was performed, as no heterogeneity was shown in heterogeneity analysis (RCTs: chi^2^ = 0.05, df = 1, *P* = 0.82, *I*^2^ = 0%; total: chi^2^ = 0.26, df = 2, *P* = 0.88, *I*^2^ = 0%).

##### Infection

The outcome of infection included superficial infection and deep infection around the wounds. Four studies with 552 patients reported on these outcomes. No significant difference was found in both subgroups of RCTs (RR = 0.61, 95%CI [0.16, 2.42], *P* = 0.48; Fig. 8) and observational studies (RR = 0.60, 95%CI [0.25, 1.43], *P* = 0.25; Fig. 8). The fixed-effects model and a combined analysis were conducted due to a lack of heterogeneity between studies. However, no significant difference was found (observational studies: chi^2^ = 0.44, df = 1, *P* = 0.51, *I*^2^ = 0%; total: chi^2^ = 0.44, df = 2, *P* = 0.80, *I*^2^ = 0%).

##### Other complications

**Fig. 9:**
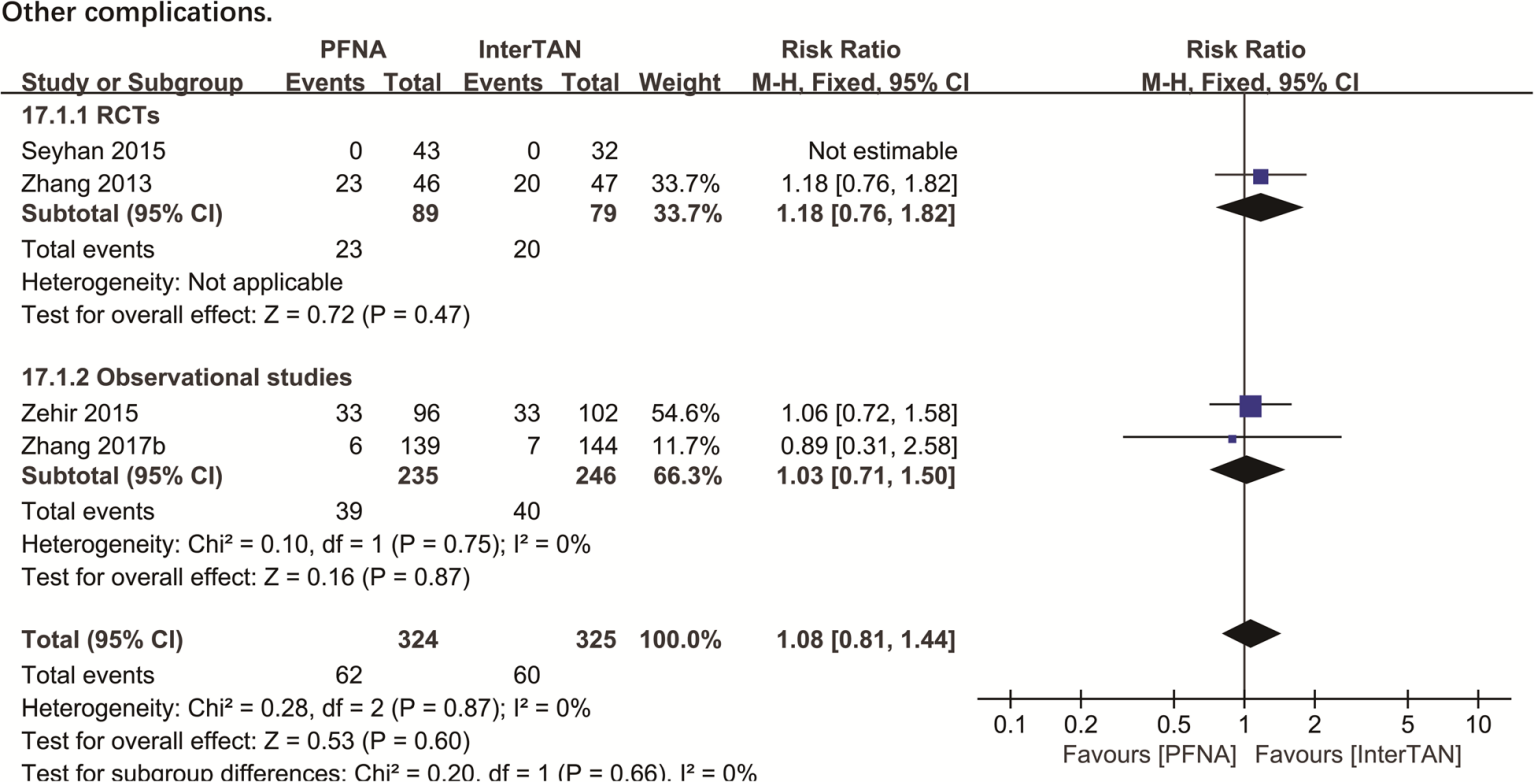
A forest plot diagram showed other complications

Other complications included deep venous thrombosis, pulmonary embolism, decompensated heart failure, urinary tract infection, pneumonia, and pressure ulcer. Four studies (*n* = 649 patients) reported on these outcomes. Overall, we found no significant difference in both subgroup analysis of RCTs (RR = 1.18, 95%CI [0.76, 1.82], *P* = 0.47; Fig. 9) and observational studies (RR = 1.03, 95%CI [0.71,1.50], *P* = 0.87; Fig. 9), which was consistent with the pooled result of combined analysis. The fixed-effects model was performed, as the heterogeneity analysis had not shown a significant difference (observational studies: chi^2^ = 0.10, df = 1, *P* = 0.75, *I*^2^ = 0%; total: chi^2^ = 0.28, df = 2, *P* = 0.87, *I*^2^ = 0%).

#### Sensitivity analysis and publication bias

We performed sensitivity analysis by iteratively excluding one study at a time to confirm the robustness of the results. All the results were robust except for the outcome of pain at the hip or thigh. When a certain study [20] resulting in heterogeneity was removed, no heterogeneity was found in the remaining studies (RCTs: chi^2^ = 0.83, df = 1, *P* = 0.36, *I*^2^ = 0%; observational studies: chi^2^ = 0.11, df = 2, *P* = 0.95, *I*^2^ = 0%; total: chi^2^ = 0.95, df = 4, *P* = 0.92, *I*^2^ = 0%; Fig S1), which could be explained by the different surgical skills of the surgeons between included studies. In addition, considering the length of follow-up time may have an impact on the complications of patients, by excluding 1 study with shorter follow-up time [22], it can be found that the heterogeneity has not changed significantly, which proves that the statistical results are reliable.

## Discussion

With the extension of human life expectancy, the elderly population, no matter with or without osteoporosis, will increase, and thus, the number of patients with intertrochanteric fractures will continue to increase. PFNA and InterTAN nails are commonly used intramedullary fixation devices to treat for intertrochanteric fractures, but there is controversy as to which one has more clinical advantages. In view of this, many patients will benefit greatly from surgical options that can lead to better clinical outcomes. Although a previous meta-analysis conducted by Ma et al. reported a comparison of clinical outcomes of PFNA and InterTAN intramedullary nails, they concluded that InterTAN nail was not worthy of being recommended as an alternative intramedullary nail in intertrochanteric fractures. However, a relatively small sample size of five studies with only 592 patients reduced its testing capability for statistical analysis [19]. Similarly, Cipololaro et al. recently summarized different studies on the treatment of hip fractures with single nails, double nails, and double integrated screws in the intramedullary nailing systems and found no significant difference between the PFNA group and the InterTAN group in long-term implant-related failures, revisions, post-operative pain, non-union, and HHS. However, of the eight studies that they included, one was a biomechanical study and the other compared TAN and PFNA; what is more, they did not perform meta-analysis while only described the statistical results and did not discuss it in depth [29]. In view of these, we remain doubtful about the relevant conclusions of this study, and we perform a rigorous meta-analysis to compare the clinical efficacy of PFNA and InterTAN. The present analysis included 9 studies with 1243 patients, which allowed us to compare more clinical outcome indicators. An updated comparison of clinical outcomes between the two nailing systems can provide additional evidence for the choice of clinical treatment options of intertrochanteric fractures.

In terms of HHS, blood loss, time to union, and length of hospital stay, the present evidence is consistent with the findings of previous studies, showing that PFNA and InterTAN are similar in these clinical outcomes. Previous study has shown that shorter operation time can be obtained with PFNA [19]. In contrast, our analysis showed no statistically significant difference for the outcome of operation time between the two intramedullary nails, as evidenced by both results of the RCT subgroup and observation subgroup analysis, such result is consistent with one new study we included published in 2017 [21]. It is not hard to understand that with the popularity of InterTAN applications and the surgeon’s familiarity with this device, the operative time may have been reduced. In addition, some other clinical outcomes were compared and analyzed, including intraoperative complications such as fractures occurred in greater trochanter, lateral cortex or femoral shaft, changes in distal interlocking position, penetration of trochanter or femoral head, postoperative union problems, hematoma, and infection, and other complications. Overall, these clinical outcomes of intertrochanteric fractures treated with these two types of intramedullary nails were similar, with no statistically significant difference.

Taking into account some categorical variables listed in this study, the current pooled data showed that PFNA has a higher risk of screw migration and cutout. Interestingly, the higher risk of high postoperative TAD and femoral head collapse in the PFNA group was also found compared with the InterTAN nail group. This may be related to InterTAN nail’s ability to provide stronger fracture compression fixation caused by the trapezoidal nail profile. Consistent with these findings, a retrospective study of 101 patients in 2018 reported that implant-related complications of PFNA for intertrochanteric fractures treatment were as high as 15.84%, of which 7 were cut out (6.93%), and 2 were secondary varus displacements (1.98%), while TAD > 25 mm and malposition of the spiral blade in the femoral head were found to be important risk factors for secondary varus displacement and screw cutout of the fracture [30, 31]. Moreover, a study involving 68 patients with trochanteric fractures using the proximal femoral nailing system showed that there were 36 patients with postoperative TAD < 25 mm, no screw displacement occurred, while the remaining 32 had TAD > 25 mm, and 7 had screw displacement (21.8%); it is worth noting that a total of 15 patients used InterTAN, and only one had a screw mobilization, while 6 (11.3%) receiving Zimmer Natural nail experienced screw mobilization [32].

Generally, for intertrochanteric fractures, especially unstable ones, it is particularly important to provide strong and stable intersegmental compression by intramedullary nails after fracture reduction. Seyhan et al. measured the postoperative fracture gap after intertrochanteric fracture treatment with InterTAN and PFNA intramedullary nails and showed that the InterTAN group significantly reduced the postoperative fracture gap compared to the PFNA group, providing greater compression and fixation capacity for the fracture end to maintain the stability of the fracture [14]. This may explain why InterTAN is superior to PFNA in clinical outcomes such as cutout, femoral head varus collapse, which is consistent with the present analysis results. Indeed, the relatively high risk of cutout or cut through of PFNA has been confirmed in multiple studies and is thought to be related to the design of the spiral blade [14, 33]. In response to such problem, bone cement-enhanced PFNA designs with perforated spiral blades may provide stronger fixation of fractured femoral head-neck ends. For example, in nine European clinics, 59 patients suffering from an osteoporotic pertrochanteric fracture were treated with the augmented PFNA, none of 59 patients undergo screw cutouts, cut through, blade displacement, implant loosening, or disruption during a mean follow-up of 4 months [34].

Postoperative pain is an element that affects patients' quality of life. Screw failure, femoral shaft fracture, and other implant failure-related complications may lead to long-term pain [35]. The results of the present analysis results are consistent with the findings of the above research, and 4 of 6 studies showed significantly reduced postoperative pain in the InterTAN nail group compared to the PFNA group [21, 23, 24, 26]. The occurrence of screw cutout, femoral shaft fracture, and femoral head varus collapse inevitably increased the probability of reoperation, which was confirmed by this study in which InterTAN nail significantly decreased the risk of reoperation compared with the PFNA. Notably, studies have shown that patients with intertrochanteric fractures undergo cutout or cut through after PFNA fixation, often requiring total hip arthroplasty (THA) to obtain better reoperation outcomes, while the effect of reintramedullary nail fixation was not satisfactory [36]. Hence, careful consideration of the surgical options is key when reoperation is performed on patients with intertrochanteric fractures.

This study has some inherent limitations. Firstly, there are relatively few RCT studies in the included studies, which may increase potential bias to a certain extent. The present results will be better convinced if more RCT studies can be included. Secondly, the follow-up time of patients was various among the included literature, which may cause the heterogeneity and bias. Although some limitations exist in the present study, the extracted data are of high quality according to the inclusion and exclusion criteria.

## Conclusions

In this meta-analysis, PFNA has not found to be superior to InterTAN nail in terms of HHS, operation time, mean length of hospital stay, time to union, union problems, intraoperative complications, hematoma, infection, and other complications. However, the present analysis reveals that patients received surgery with InterTAN nail had lower risk of the screw migration, pain at thigh or hip, cutout, varus collapse of the femoral head, femoral shaft fracture, and reoperation, it is concluded that InterTAN may be recommended as a preferred clinical treatment for intertrochanteric fractures in comparison to PFNA.

## Supplementary information

**Additional file 1:** Supplemental List 1.

**Additional file 2:** **Figure S1.** A forest plot diagram showed pain at thigh or hip when the study [20] was removed.

## Data Availability

Not applicable.

## Abbreviations

PFNA: Proximal femoral nail anti-rotation
RS: Retrospective study
RCTs: Randomized control trials
HHS: Harris Hip Score
RR: The relative risk
MD: The mean difference
AO/OTA: Arbeitsge-meinschaft für Osteosynthesefragen/Orthopaedic Trauma Association
