# Supplementary material for: Comparison of clinical outcomes with proximal femoral nail anti-rotation versus InterTAN nail for intertrochanteric femoral fractures: a meta-analysis
Source: J Orthop Surg Res. 2020 Oct 29;15:500. doi: 10.1186/s13018-020-02031-8 (PMC7596936; doi:10.1186/s13018-020-02031-8)
Supplement: Supplementary file 1 — Additional file 1: Supplemental List 1. [file 13018_2020_2031_MOESM1_ESM.docx]

The search terms

((((((((((((((hip fracture[MeSH Terms]) OR femoral fractures) OR femoral intertrochanteric fracture) OR intertrochanteric femoral fracture) OR intertrochanteric fracture) OR intertrochanteric fracture of femur) OR trochanteric fractures) OR trochanteric fractures) OR intertrochanteric femur fracture) OR intertrochanteric hip fractures) OR extracapsular hip fractures) OR extracapsular femoral fractures)) AND ((((((((Fracture Fixation, Intramedullary[MeSH Terms]) OR Fracture Fixation, Internal) OR PFNA) OR Proximal femoral nail antirotation) OR single screw cephalomedullary nail) OR PFNAII) OR proximal femoral nail antirotation-Asia) OR Helical Blade)) AND (((((InterTAN nail) OR InterTAN) OR Integrated 2 screw derotation cephalomedullary device) OR Integrated Lag Screws) OR Intertrochanteric Antegrade Nail)
