# Supplementary figures and images for: Comparison of clinical outcomes with proximal femoral nail anti-rotation versus InterTAN nail for intertrochanteric femoral fractures: a meta-analysis
Source: J Orthop Surg Res. 2020 Oct 29;15:500. doi: 10.1186/s13018-020-02031-8 (PMC7596936; doi:10.1186/s13018-020-02031-8)

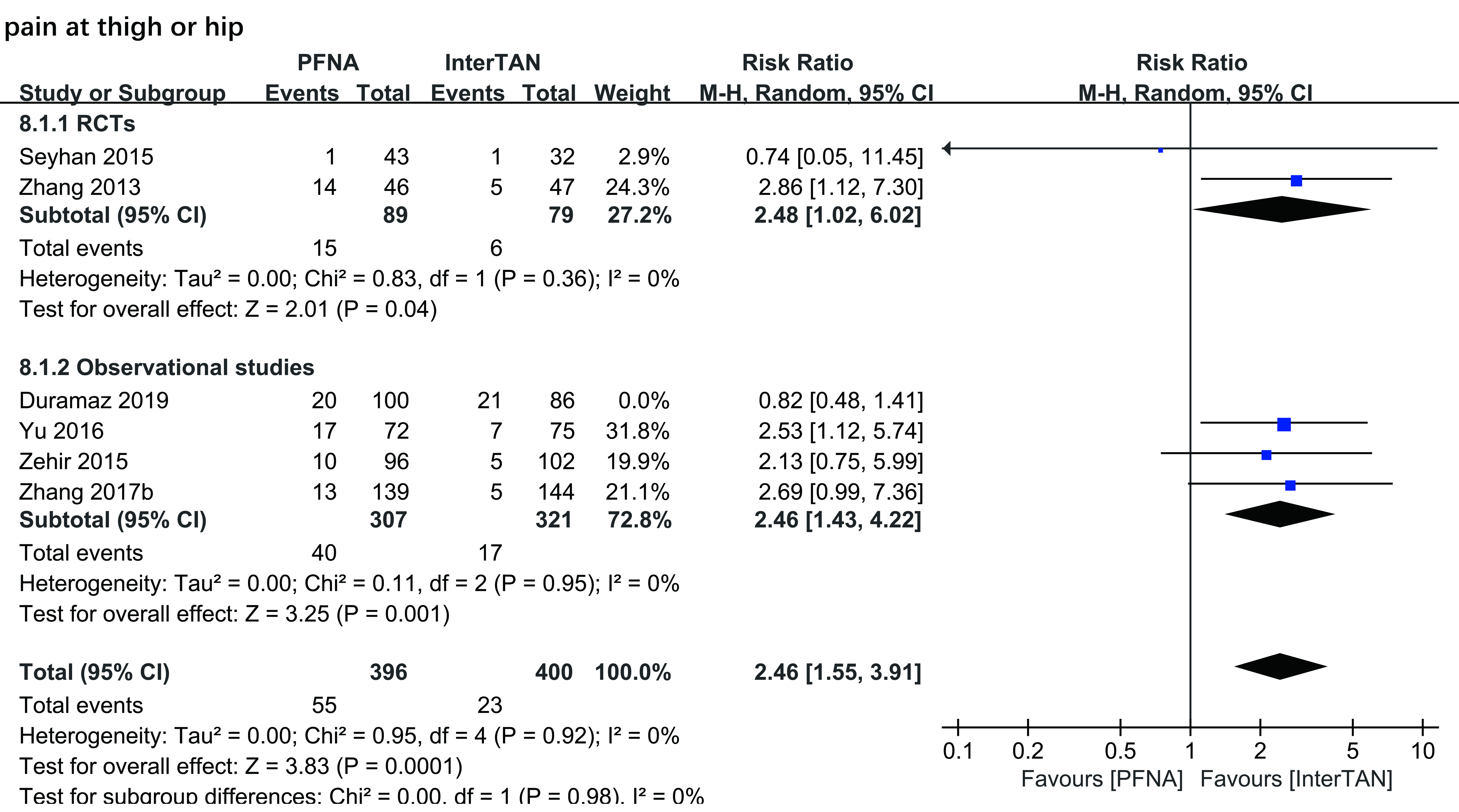

Supplement: Supplementary file 2 — Additional file 2: Figure S1. A forest plot diagram showed pain at thigh or hip when the study [20] was removed. [file 13018_2020_2031_MOESM2_ESM.tif]
